# Derived metabolic indices and poor early response to corticosteroids in adults with primary immune thrombocytopenia: a retrospective cohort study

**DOI:** 10.3389/fendo.2026.1981987

**Published:** 2026-10-28

**Authors:** Xin Zhou, Guangzhen Lu, Jiarui Song, Xiaowen Tian, Lechang Zhang, Ningning Shan

**Affiliations:** 1 Department of Hematology, Shandong Provincial Hospital Affiliated to Shandong First Medical University, Jinan, Shandong, China; 2 Department of Hematology, The Second Qilu Hospital of Shandong University, Jinan, Shandong, China; 3 Department of Cardiology, Shandong Provincial Hospital Affiliated to Shandong First Medical University, Jinan, Shandong, China; 4 Department of Gastroenterology, Shandong Provincial Hospital Affiliated to Shandong First Medical University, Jinan, Shandong, China

**Keywords:** atherogenic index of plasma, corticosteroids, early treatment response, immune thrombocytopenia, triglyceride glucose index

## Abstract

**Objective:**

To evaluate the associations of the pretreatment atherogenic index of plasma (AIP), triglyceride–glucose index (TyG), and log-transformed blood urea nitrogen-to-high-density lipoprotein cholesterol ratio (logBHR) with poor early response to corticosteroid therapy in adults with primary immune thrombocytopenia (ITP).

**Methods:**

This single-center retrospective cohort study included 259 patients with ITP. Multivariable logistic regression was used to evaluate the associations of AIP, TyG, and logBHR with early treatment response. Three-knot restricted cubic spline models were used to examine the functional forms of the associations. Receiver operating characteristic curve analysis was performed to explore the ability of each derived index to discriminate early treatment response.

**Results:**

Among the 259 patients, 66 (25.5%) had a poor early response. AIP, TyG, and logBHR levels were higher in nonresponders than in responders. After adjustment for the prespecified covariates, the ORs for day-7 nonresponse per one-unit increase in AIP, TyG, and logBHR were 5.726 (95% CI, 1.711–20.150), 2.105 (95% CI, 1.194–3.780), and 2.331 (95% CI, 1.093–5.075), respectively. Compared with the lowest quartile, the adjusted ORs for the highest quartile were 3.247 (95% CI, 1.384–8.105), 2.363 (95% CI, 1.051–5.531), and 4.272 (95% CI, 1.716–11.560), respectively. Restricted cubic spline analyses showed overall associations of AIP, TyG, and logBHR with day-7 nonresponse (*P* for overall association = 0.021, 0.034, and 0.047, respectively), with no clear evidence of nonlinearity (*P* for nonlinearity = 0.495, 0.353, and 0.142, respectively). The adjusted models incorporating the corresponding indices yielded AUCs ranging from 0.658 to 0.675.

**Conclusions:**

To our knowledge, this is the first study linking higher pretreatment AIP, TyG, and logBHR to day-7 nonresponse to corticosteroid therapy in adults with primary ITP. These exploratory findings require prospective validation before clinical application.

## 1 Introduction

Immune thrombocytopenia (ITP) is an acquired autoimmune disorder characterized by increased platelet destruction and impaired platelet production resulting from loss of immune tolerance. Its clinical presentation is highly heterogeneous, ranging from asymptomatic thrombocytopenia to life-threatening bleeding (1, 2). Thrombopoietin receptor agonists (TPO-RAs) have substantially expanded treatment options for adults and children with ITP, particularly those requiring treatment beyond first-line therapy (3). Corticosteroids remain the most commonly used first-line treatment for adults with newly diagnosed ITP (4). However, early responses to corticosteroid therapy vary considerably among patients, and a substantial proportion show an inadequate initial response or experience rapid relapse. This may increase exposure to corticosteroid-related adverse effects and delay the timely initiation of subsequent treatment strategies (5). Therefore, early identification of patients at high risk of a poor response is clinically important for optimizing individualized treatment decisions, minimizing ineffective corticosteroid exposure, and improving patient outcomes.

Conventional metabolic and inflammatory markers have been investigated in ITP. Studies of lipid measures and C-reactive protein have examined their associations with disease severity and treatment outcomes (6, 7). Blood cell–derived indices have also been evaluated in relation to corticosteroid response, with varying findings (8). More broadly, indices combining routine biochemical measurements have attracted increasing interest in clinical research (9). This approach provides a basis for exploring whether combinations of readily available biochemical measures are associated with early treatment response in ITP.

The atherogenic index of plasma (AIP), calculated from triglyceride (TG) and high-density lipoprotein cholesterol (HDL-C) levels, reflects the characteristics of the atherogenic lipoprotein profile and the degree of lipid metabolic disturbance (10, 11). The triglyceride–glucose index (TyG), derived from fasting TG and glucose levels, is a reliable surrogate marker of insulin resistance and glucose–lipid metabolic dysfunction (12, 13).The blood urea nitrogen-to-high-density lipoprotein cholesterol ratio (BHR) integrates blood urea nitrogen (BUN) and HDL-C and may reflect nitrogen metabolism, renal function-related alterations, and lipid metabolic abnormalities. BHR has also been associated with systemic inflammation and outcomes across a range of diseases (14). Evidence linking metabolic regulation to immune function provides a biological rationale for investigating metabolic indices in relation to corticosteroid response in ITP (15). Plasma metabolomic profiling has also identified differences associated with glucocorticoid responsiveness in patients with chronic ITP (16). These findings provide a biological rationale for investigating these indices in relation to corticosteroid response, although their associations with early treatment response in ITP remain unclear.Together, these observations support the hypothesis that higher pretreatment AIP, TyG, and BHR are associated with greater odds of nonresponse at day 7.

Accordingly, this study evaluated the associations of AIP, TyG, and BHR with poor early response to corticosteroid therapy in adults with ITP and further examined their dose–response relationships and consistency across subgroups.

## 2 Methods

### 2.1 Study design and participants

This single-center retrospective observational study consecutively enrolled adults with primary ITP who were hospitalized in the Department of Hematology at Shandong Provincial Hospital Affiliated to Shandong First Medical University between January 2019 and February 2026. Primary ITP was diagnosed according to the American Society of Hematology guidelines (4), based on a peripheral blood platelet count of <100 × 10^9^/L and systematic exclusion of secondary thrombocytopenia and other causes of thrombocytopenia. The diagnostic assessment included a review of medical history, medication exposure, infection history, and family history, together with physical examination. Laboratory investigations included complete blood counts, peripheral blood smear examination, liver and renal function tests, coagulation studies, viral testing and autoimmune screening, including antinuclear antibodies. Bone marrow aspiration was performed at admission before treatment initiation to assess hematopoiesis and aid in the differential diagnosis of other hematologic disorders. Megakaryocyte subtypes were classified according to established morphological criteria (17), with the corresponding data retrospectively extracted from routine bone marrow examination reports in the hospital medical records. (17).

The inclusion criteria were as follows: (1) age ≥18 years; (2) newly diagnosed primary ITP; (3) receipt of a corticosteroid-containing first-line regimen, including prednisone, methylprednisolone, or high-dose dexamethasone (HD-DXM); (4) availability of complete baseline clinical and laboratory data; and (5) availability of an early treatment-response assessment. The exclusion criteria were as follows: (1) secondary ITP associated with autoimmune disease, infection, lymphoproliferative disease, or drug-induced thrombocytopenia; (2) previous receipt of second-line or later-line ITP treatment; (3) missing key clinical variables; and (4) incomplete treatment or response-assessment data that precluded determination of the early treatment response.

At our center, initial corticosteroid treatment was primarily based on dexamethasone. The study was approved by the Medical Ethics Committee of Shandong Provincial Hospital Affiliated to Shandong First Medical University and was conducted in accordance with the principles of the Declaration of Helsinki. Given the retrospective study design, all patient data were anonymized.

### 2.2 Outcome definition

The primary outcome was day-7 nonresponse under corticosteroid-containing first-line treatment. Treatment response was assessed on day 7 in accordance with the Chinese Guideline for the Diagnosis and Treatment of Adult Primary Immune Thrombocytopenia (2025) (18).Complete response (CR) was defined as a platelet count of ≥100 × 10^9^/L with no bleeding. Response (R) was defined as a platelet count of ≥30 × 10^9^/L, at least a twofold increase from baseline, and no bleeding. No response (NR) was defined as failure to meet the criteria for CR or R, or the persistence of clinically significant bleeding. For the present analysis, patients who achieved CR or R were classified as the early-response group, whereas those with NR were classified as the poor-early-response group.

### 2.3 Data preprocessing and derived indices

Missing values were imputed using the random forest method implemented in the mice package. Five imputed datasets were generated, and the first completed dataset was used for subsequent analyses. After imputation, selected laboratory variables were winsorized at the 1st and 99th percentiles to limit the influence of extreme values. Values below or above these percentiles were replaced with the corresponding percentile values. The derived indices were then calculated from the processed laboratory data.

The metabolic indices were calculated using baseline laboratory measurements obtained at the initial hospitalization before treatment. The AIP was defined as the base-10 logarithm of the ratio of TG to HDL-C: AIP = log10(TG/HDL-C), where both TG and HDL-C were expressed in mmol/L. The TyG, a marker of glucose and lipid metabolic status, was calculated as follows: TyG = ln [TG (mg/dL) × FPG (mg/dL)/2]. To ensure consistency in the calculation, the clinically measured TG and fasting plasma glucose (FPG) values were converted to mg/dL before the TyG index was calculated. The BHR was calculated by dividing BUN by HDL-C: BHR = BUN/HDL-C. Because BHR had a right-skewed distribution, it was analyzed on the natural-log scale [logBHR].

### 2.4 Covariate selection and model adjustment

AIP, TyG, and logBHR were the primary exposures of interest. Other baseline variables were used to characterize the study population, adjust the regression models, or define exploratory subgroups. Logistic regression models were used to evaluate the associations of AIP, TyG, and logBHR with the outcome and to estimate odds ratios (ORs) and 95% confidence intervals (CIs). Covariates were prespecified based on their clinical relevance, evidence from previous studies, and data availability (19–21). Age and sex were included as basic demographic covariates, whereas the available baseline viral markers, including Epstein–Barr virus (EBV) status, hepatitis B surface antigen (HBsAg) status, and hepatitis B e antibody (HBeAb) status, were included in the fully adjusted model. Model 1 was unadjusted. Model 2 was adjusted for age and sex. Model 3 was additionally adjusted for EBV, HBsAg, and HBeAb status. AIP, TyG, and logBHR were examined separately as continuous and categorical variables, and their associations with the odds of poor early response were expressed as ORs with 95% CIs.

### 2.5 Statistical analysis

Continuous variables were assessed for normality using the Shapiro–Wilk test. They were summarized as mean (SD) or median (IQR) and compared using Welch’s t test or the Kruskal–Wallis test, respectively. Categorical variables were presented as numbers and percentages and compared using chi-square tests, with continuity correction where applicable, or Fisher’s exact test.

AIP, TyG, and logBHR were analyzed separately as continuous variables and in quartiles. ORs and 95% CIs were reported per one-unit increase for continuous analyses and relative to the lowest quartile for categorical analyses. Trends across quartiles were tested by assigning participants the median value of their quartile and entering this value as a continuous variable. Restricted cubic spline models assessed potential nonlinearity, using three knots at the 10th, 50th, and 90th percentiles, the median as the reference, and the Model 3 covariates. Overall associations and nonlinearity were evaluated using Wald chi-square tests. Exploratory subgroup analyses used unadjusted logistic regression. Indices were standardized before stratification, and ORs were reported per one-SD increase. Differences in associations between subgroups were assessed using interaction terms.

ROC curves were constructed from the predicted probabilities of the models containing each index and the Model 3 covariates. Discrimination was evaluated using AUCs with 95% CIs calculated by DeLong’s method. Probability thresholds were selected using the Youden index, with corresponding sensitivity, specificity, accuracy, and predictive values reported. Performance was assessed in the same dataset used to fit the models. All tests were two-sided, and *P* < 0.05 was considered statistically significant. Analyses were performed using R software, version 4.4.1.

## 3 Results

### 3.1 Study population and baseline characteristics

A total of 336 patients were screened. After application of the eligibility criteria, 259 adults with primary ITP who received first-line corticosteroid therapy were included in the analysis (**Figure 1**). Participants were aged 18–84 years, with a median age of 51 years (IQR, 39–62 years); 164 (63.3%) were women. Of these, 193 patients (74.5%) achieved an early treatment response by day 7 and were classified as the early-response group, whereas 66 (25.5%) did not meet the prespecified response criteria and were classified as the poor-early-response group. Baseline demographic, clinical, and laboratory characteristics are presented according to day-7 treatment response in **Table 1**.

**Figure 1:**
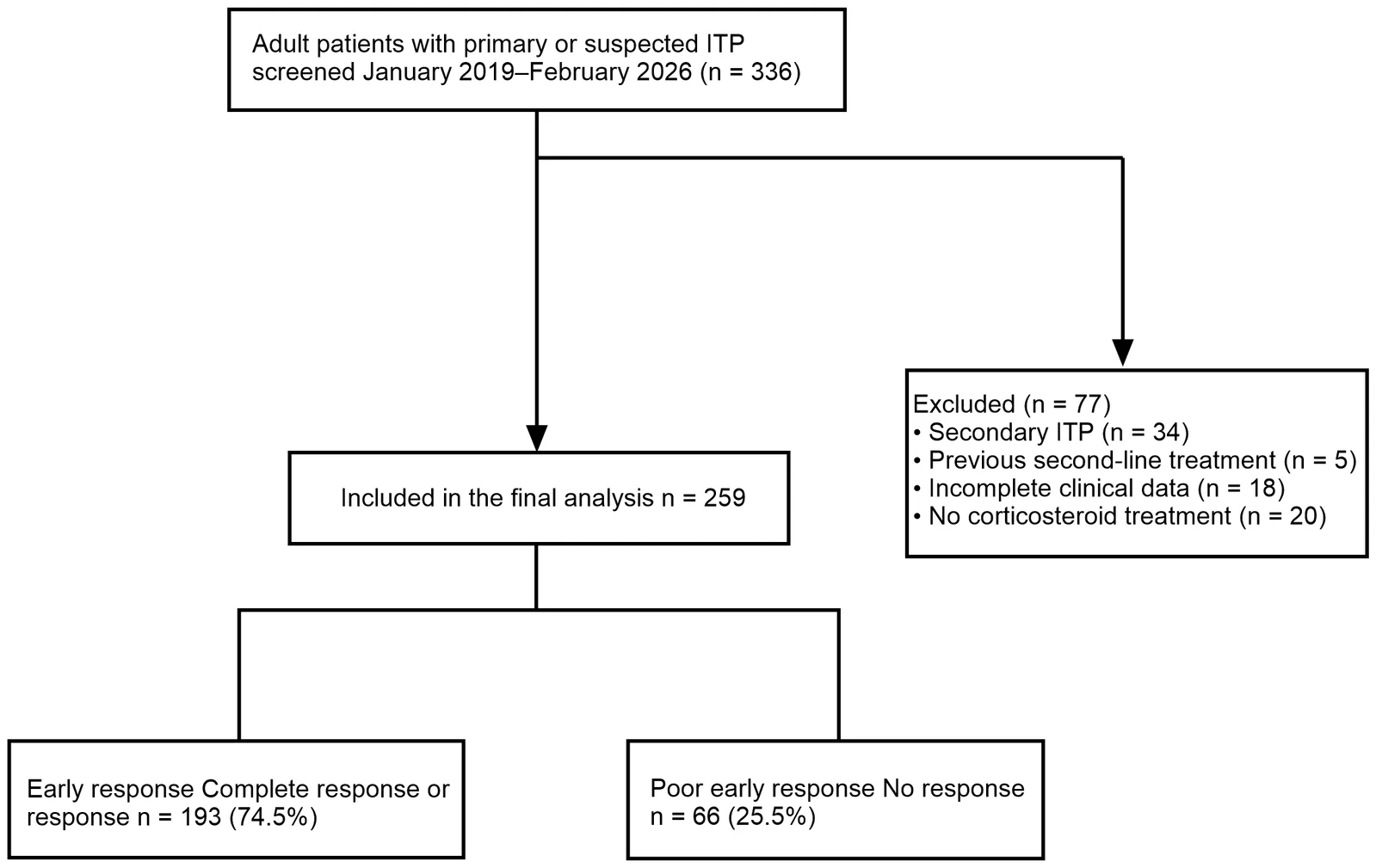
Flow diagram of patient selection and study-group assignment.

**Table 1:** Clinical and laboratory characteristics according to early treatment response.

Characteristic	Overall (n = 259)	Early-response group (n = 193)	Poor-response group (n = 66)	*P* value
Demographic characteristics				
Age, years	51.00 [39.00, 62.00]	50.00 [37.00, 62.00]	54.50 [41.25, 64.75]	0.118
Female, n (%)	164 (63.3)	121 (62.7)	43 (65.2)	0.834
Male, n (%)	95 (36.7)	72 (37.3)	23 (34.8)	
Hematological characteristics				
Baseline PLT, ×10^9^/L	13.00 [4.50, 26.50]	13.00 [5.00, 26.00]	12.00 [4.00, 26.75]	0.940
WBC, ×10^9^/L	7.08 [5.26, 9.62]	7.08 [5.14, 9.64]	7.02 [5.51, 9.52]	0.951
HGB, g/L	125.00 [111.00, 137.00]	124.00 [111.00, 135.00]	127.00 [111.50, 140.00]	0.315
ALC, ×10^9^/L	1.44 [0.92, 2.04]	1.43 [0.86, 2.03]	1.46 [1.00, 2.04]	0.703
ANC, ×10^9^/L	4.99 [3.12, 7.11]	5.03 [3.12, 7.34]	4.55 [3.16, 6.65]	0.573
MPV, fL	12.10 [10.70, 13.50]	11.90 [10.60, 13.00]	12.75 [11.17, 14.07]	0.007
P-LCR, %	41.90 [34.80, 48.20]	41.10 [34.10, 47.60]	45.20 [36.27, 50.93]	0.039
Immunological and infection markers				
ANA-IgG positivity, n (%)	53 (20.5)	42 (21.8)	11 (16.7)	0.478
Antiplatelet antibody positivity, n (%)	12 (4.6)	4 (2.1)	8 (12.1)	0.003
T-SPOT.TB positivity, n (%)	17 (6.6)	11 (5.7)	6 (9.1)	0.501
EBV positivity, n (%)	29 (11.2)	19 (9.8)	10 (15.2)	0.340
HBcAb positivity, n (%)	98 (37.8)	64 (33.2)	34 (51.5)	0.012
HBeAb positivity, n (%)	59 (22.8)	39 (20.2)	20 (30.3)	0.129
HBsAg positivity, n (%)	16 (6.2)	9 (4.7)	7 (10.6)	0.151
Coagulation parameters				
D-dimer, mg/L	0.40 [0.24, 0.86]	0.40 [0.22, 0.68]	0.50 [0.30, 1.40]	0.004
PT, s	11.20 [10.60, 12.30]	11.20 [10.70, 12.10]	11.40 [10.50, 12.50]	0.860
APTT, s	28.90 [26.00, 31.60]	29.00 [26.00, 31.60]	28.90 [26.02, 31.70]	0.926
FIB, g/L	2.93 [2.49, 3.52]	2.84 [2.47, 3.49]	3.01 [2.55, 3.59]	0.238
Liver and renal function				
ALT, U/L	19.00 [13.00, 31.00]	20.00 [13.00, 31.00]	17.00 [14.00, 27.75]	0.983
AST, U/L	20.00 [15.50, 25.00]	20.00 [15.00, 25.00]	19.50 [16.00, 25.00]	0.765
ALB, g/L	40.40 [37.20, 43.35]	40.60 [37.20, 43.40]	40.15 [37.25, 43.00]	0.788
TBIL, μmol/L	12.64 [9.80, 16.09]	12.65 [9.70, 15.90]	12.23 [10.05, 17.27]	0.416
BUN, mmol/L	5.60 [4.50, 6.60]	5.50 [4.40, 6.40]	5.65 [4.93, 7.05]	0.098
Creatinine, μmol/L	53.70 [45.65, 65.35]	53.40 [45.60, 65.80]	54.50 [46.62, 64.22]	0.728
Lipid and glucose metabolism				
TG, mmol/L	1.09 [0.84, 1.50]	1.05 [0.83, 1.46]	1.20 [0.92, 1.65]	0.037
TC, mmol/L	4.75 [4.05, 5.55]	4.74 [4.02, 5.40]	4.95 [4.13, 5.76]	0.177
HDL-C, mmol/L	1.37 [1.19, 1.62]	1.43 [1.22, 1.65]	1.31 [1.10, 1.49]	0.012
LDL-C, mmol/L	2.92 [2.30, 3.68]	3.04 [2.26, 4.03]	2.82 [2.49, 3.40]	0.761
Glucose, mmol/L	5.48 [4.84, 6.44]	5.48 [4.74, 6.38]	5.51 [4.94, 6.54]	0.239
GA, %	13.93 [12.74, 15.46]	13.59 [12.50, 15.00]	15.32 [13.32, 16.84]	<0.001
Bone marrow characteristics				
Total megakaryocytes, n	113.00 [36.00, 214.00]	113.00 [37.00, 210.00]	110.00 [34.25, 244.50]	0.914
Immature megakaryocytes, n	2.00 [1.00, 10.00]	2.00 [1.00, 7.00]	3.00 [1.00, 12.00]	0.040
Granular megakaryocytes, n	82.00 [26.00, 165.00]	83.00 [27.00, 160.00]	81.00 [19.00, 174.00]	0.853
Granular megakaryocytes, %	80 [66, 88]	80 [64, 87]	84 [75, 88]	0.024
Platelet-producing megakaryocytes, n	4.00 [1.00, 13.00]	4.00 [1.00, 12.00]	4.50 [2.00, 20.50]	0.456
Derived metabolic indices				
AIP	−0.09 (0.24)	−0.12 (0.24)	−0.02 (0.23)	0.003
TyG	8.49 [8.19, 8.82]	8.43 [8.16, 8.76]	8.66 [8.27, 8.91]	0.009
BHR	3.93 [3.16, 5.06]	3.84 [3.08, 4.72]	4.70 [3.63, 5.60]	0.001
logBHR	1.37 [1.15, 1.62]	1.35 [1.13, 1.55]	1.55 [1.29, 1.72]	0.001
AIP quartile, n (%)				0.075
Q1	65 (25.1)	55 (28.5)	10 (15.2)	
Q2	65 (25.1)	49 (25.4)	16 (24.2)	
Q3	64 (24.7)	47 (24.4)	17 (25.8)	
Q4	65 (25.1)	42 (21.8)	23 (34.8)	
TyG quartile, n (%)				0.041
Q1	65 (25.1)	52 (26.9)	13 (19.7)	
Q2	65 (25.1)	54 (28.0)	11 (16.7)	
Q3	64 (24.7)	46 (23.8)	18 (27.3)	
Q4	65 (25.1)	41 (21.2)	24 (36.4)	
logBHR quartile, n (%)				0.003
Q1	65 (25.1)	57 (29.5)	8 (12.1)	
Q2	65 (25.1)	51 (26.4)	14 (21.2)	
Q3	64 (24.7)	46 (23.8)	18 (27.3)	
Q4	65 (25.1)	39 (20.2)	26 (39.4)	

Data are presented as mean (SD), median [IQR], or n (%), as appropriate. P values compare the early-response and poor-response groups using the independent-samples t test, Mann–Whitney U test, Pearson’s chi-square test, or Fisher’s exact test, as appropriate. Between-group comparisons in this table are descriptive.

AIP, atherogenic index of plasma; ALB, albumin; ALC, absolute lymphocyte count; ALT, alanine aminotransferase; ANA-IgG, antinuclear antibodies of the immunoglobulin G class; ANC, absolute neutrophil count; APTT, activated partial thromboplastin time; AST, aspartate aminotransferase; BHR, blood urea nitrogen-to-high-density lipoprotein cholesterol ratio; BUN, blood urea nitrogen; EBV, Epstein–Barr virus; FIB, fibrinogen; GA, glycated albumin; HBcAb, hepatitis B core antibody; HBeAb, hepatitis B e antibody; HBsAg, hepatitis B surface antigen; HDL-C, high-density lipoprotein cholesterol; HGB, hemoglobin; IQR, interquartile range; LDL-C, low-density lipoprotein cholesterol; logBHR, natural logarithm of the blood urea nitrogen-to-high-density lipoprotein cholesterol ratio; MPV, mean platelet volume; P-LCR, platelet large cell ratio; PLT, platelet count; PT, prothrombin time; Q1–Q4, first to fourth quartiles; SD, standard deviation; TBIL, total bilirubin; TC, total cholesterol; TG, triglycerides; T-SPOT.TB, a T-cell-based interferon-γ release assay for tuberculosis; TyG, triglyceride–glucose index; WBC, white blood cell count.

### 3.2 Association between derived indices and poor early response

The direction of the associations was consistent across all three models, with the fully adjusted Model 3 considered the primary analysis (**Table 2**). When modeled as continuous variables, higher AIP, TyG, and logBHR values were each significantly associated with greater odds of poor early response. For each one-unit increase in AIP, the OR for day-7 nonresponse was 5.726 (95% CI, 1.711–20.150; *P* = 0.005). The corresponding ORs were 2.105 (95% CI, 1.194–3.780; *P* = 0.011) for TyG and 2.331 (95% CI, 1.093–5.075; *P* = 0.030) for logBHR.

**Table 2:** Associations of AIP, TyG, and logBHR with poor early corticosteroid response across three logistic regression models.

Characteristic	Model 1			Model 2			Model 3		
	OR	95% CI	P-value	OR	95% CI	P-value	OR	95% CI	P-value
**AIP**	5.554	1.711, 18.94	0.005	5.422	1.658, 18.61	0.006	5.726	1.711, 20.15	0.005
*Quartile median, per unit increase*	6.65	1.596, 29.02	0.01	6.408	1.517, 28.29	0.013	7.183	1.650, 32.87	0.01
AIP_group									
Q1(≤-0.272)	—	—		—	—		—	—	
Q2(−0.272~−0.086)	1.796	0.755, 4.448	0.192	1.822	0.759, 4.551	0.185	2	0.818, 5.109	0.135
Q3(−0.086~0.0612)	1.989	0.843, 4.905	0.122	1.989	0.839, 4.928	0.125	2.087	0.865, 5.276	0.108
Q4(>0.0612)	3.012	1.325, 7.263	0.01	2.974	1.295, 7.238	0.012	3.247	1.384, 8.105	0.009
**TyG**	2.102	1.213, 3.707	0.009	1.998	1.146, 3.543	0.016	2.105	1.194, 3.780	0.011
*Quartile median, per unit increase*	2.46	1.255, 4.893	0.009	2.328	1.180, 4.661	0.016	2.442	1.220, 4.971	0.012
TyG_group									
Q1(≤8.19)	—	—		—	—		—	—	
Q2(8.19~8.49)	0.815	0.330, 1.983	0.652	0.811	0.327, 1.978	0.644	0.885	0.351, 2.212	0.794
Q3(8.49~8.82)	1.565	0.696, 3.599	0.282	1.6	0.707, 3.704	0.262	1.757	0.756, 4.205	0.195
Q4(>8.82)	2.341	1.077, 5.272	0.035	2.2	1.001, 5.001	0.053	2.363	1.051, 5.531	0.041
**logBHR**	2.584	1.289, 5.316	0.008	2.562	1.221, 5.504	0.014	2.331	1.093, 5.075	0.03
*Quartile median, per unit increase*	5.543	2.242, 14.23	<0.001	5.598	2.166, 15.04	<0.001	5.066	1.933, 13.77	0.001
logBHR_group									
Q1(<1.15)	—	—		—	—		—	—	
Q2(1.15~1.37)	1.956	0.773, 5.255	0.165	1.995	0.780, 5.415	0.158	1.795	0.689, 4.931	0.238
Q3(1.37~1.62)	2.788	1.144, 7.333	0.029	2.895	1.168, 7.728	0.026	2.819	1.130, 7.567	0.031
Q4(>1.62)	4.75	2.022, 12.23	<0.001	4.825	1.967, 12.92	<0.001	4.272	1.716, 11.56	0.003

AIP, atherogenic index of plasma; CI, confidence interval; EBV, Epstein–Barr virus; HBeAb, hepatitis B e antibody; HBsAg, hepatitis B surface antigen; logBHR, natural logarithm of the blood urea nitrogen-to-high-density lipoprotein cholesterol ratio; OR, odds ratio; Q1–Q4, first to fourth quartiles; TyG, triglyceride–glucose index.

Model 1 was unadjusted. Model 2 was adjusted for age and sex. Model 3 was additionally adjusted for EBV, HBsAg, and HBeAb status. For continuous-variable analyses, ORs are reported per one-unit increase in each index. A one-unit increase in logBHR corresponds to an e-fold increase in BHR (approximately 2.72 times its original value).

In the quartile-based analyses, patients in the highest quartile (Q4) of each index had significantly greater odds of poor early response than those in the lowest quartile (Q1). In the fully adjusted model, the ORs for Q4 versus Q1 were 3.247 (95% CI, 1.384–8.105; *P* = 0.009) for AIP, 2.363 (95% CI, 1.051–5.531; *P* = 0.041) for TyG, and 4.272 (95% CI, 1.716–11.560; *P* = 0.003) for logBHR. Significant linear trends across quartiles were also observed for all three indices(all *P* for trend < 0.05). Complete logistic regression results, including the ORs, 95% CIs, and *P* values for all variables included in each model, are provided in **Supplementary Tables**.

### 3.3 Restricted cubic spline analyses

Higher baseline AIP, TyG, and logBHR levels were associated with higher odds of day-7 nonresponse (*P* for overall association = 0.021, 0.034, and 0.047, respectively; **Figures 2A–C**). No significant evidence of nonlinearity was found for any of the three indices (*P* for nonlinearity = 0.495, 0.353, and 0.142, respectively).

**Figure 2:**
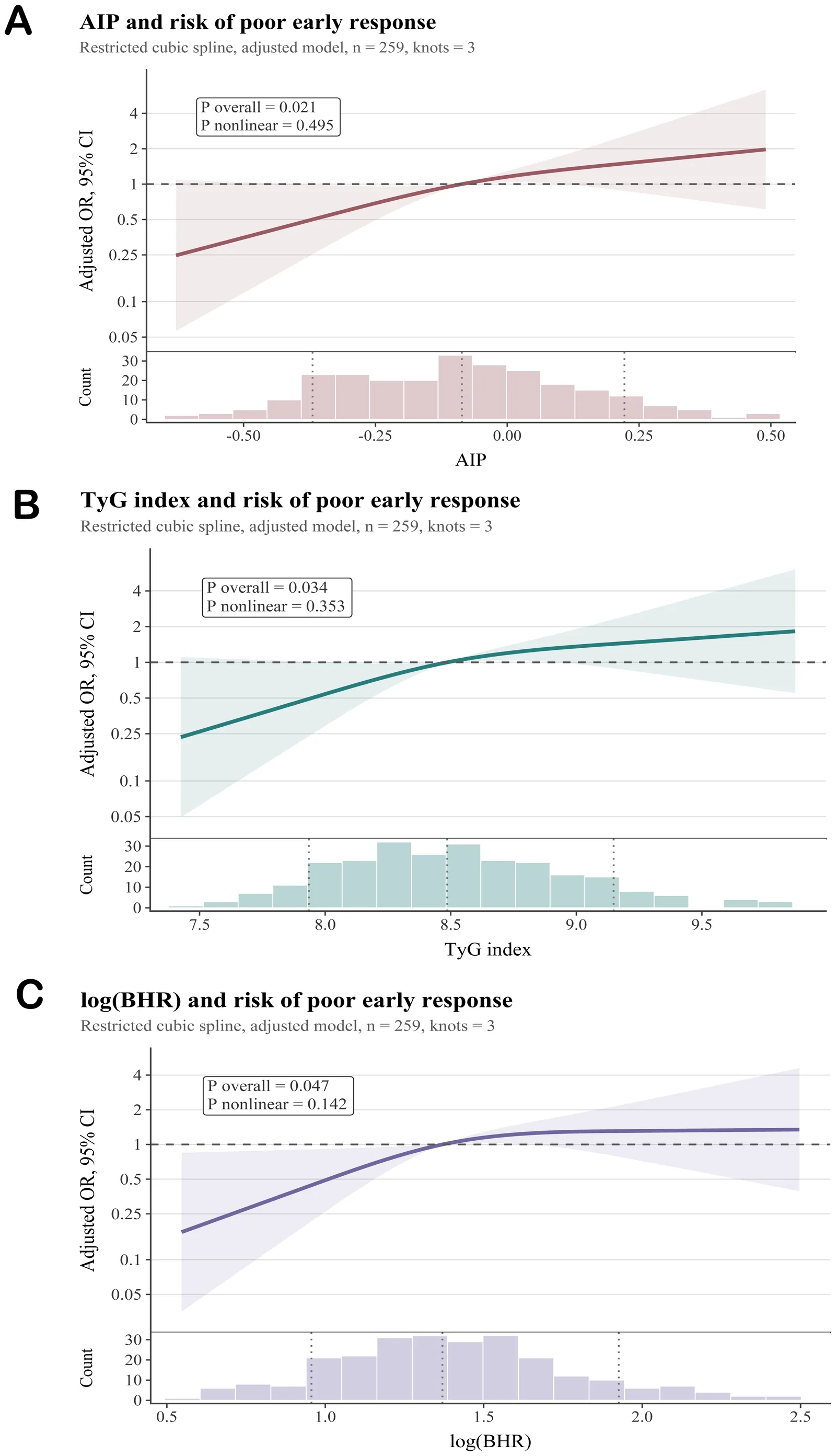
Dose–response associations of the derived metabolic indices with poor early response to corticosteroid therapy. Restricted cubic spline logistic regression models show the adjusted associations of **(A)** AIP, **(B)** TyG, and **(C)** logBHR with the odds of poor early response at day 7 in adults with primary immune thrombocytopenia. Three knots were placed at the 10th, 50th, and 90th percentiles of each index, with the median used as the reference value. Solid lines represent adjusted odds ratios, shaded areas indicate 95% confidence intervals, and histograms show the distributions of the corresponding indices. Models were adjusted for age, sex, EBV status, HBsAg status, and HBeAb status. AIP, atherogenic index of plasma; CI, confidence interval; EBV, Epstein–Barr virus; HBeAb, hepatitis B e antibody; HBsAg, hepatitis B surface antigen; logBHR [log(BHR)], natural logarithm of the blood urea nitrogen-to-high-density lipoprotein cholesterol ratio; OR, odds ratio; TyG, triglyceride–glucose index.

### 3.4 Subgroup analysis

The associations of AIP, TyG, and logBHR with day-7 nonresponse were generally consistent in direction across the examined subgroups (**Figures 3A–C**). Subgroups were defined by sex, granular megakaryocyte proportion, ANA-related measures (ANA titer, ANA status, and ANA-IgG status), antiplatelet antibody status, T-SPOT.TB results, and viral markers. Tests for interaction were not statistically significant across most prespecified subgroups for AIP or TyG. Similarly, no statistically significant interactions were observed across most subgroups for logBHR. However, a significant interaction was observed when patients were stratified using a granular megakaryocyte proportion cutoff of 0.8 (*P* for interaction = 0.036; **Figures 3A–C**).

**Figure 3:**
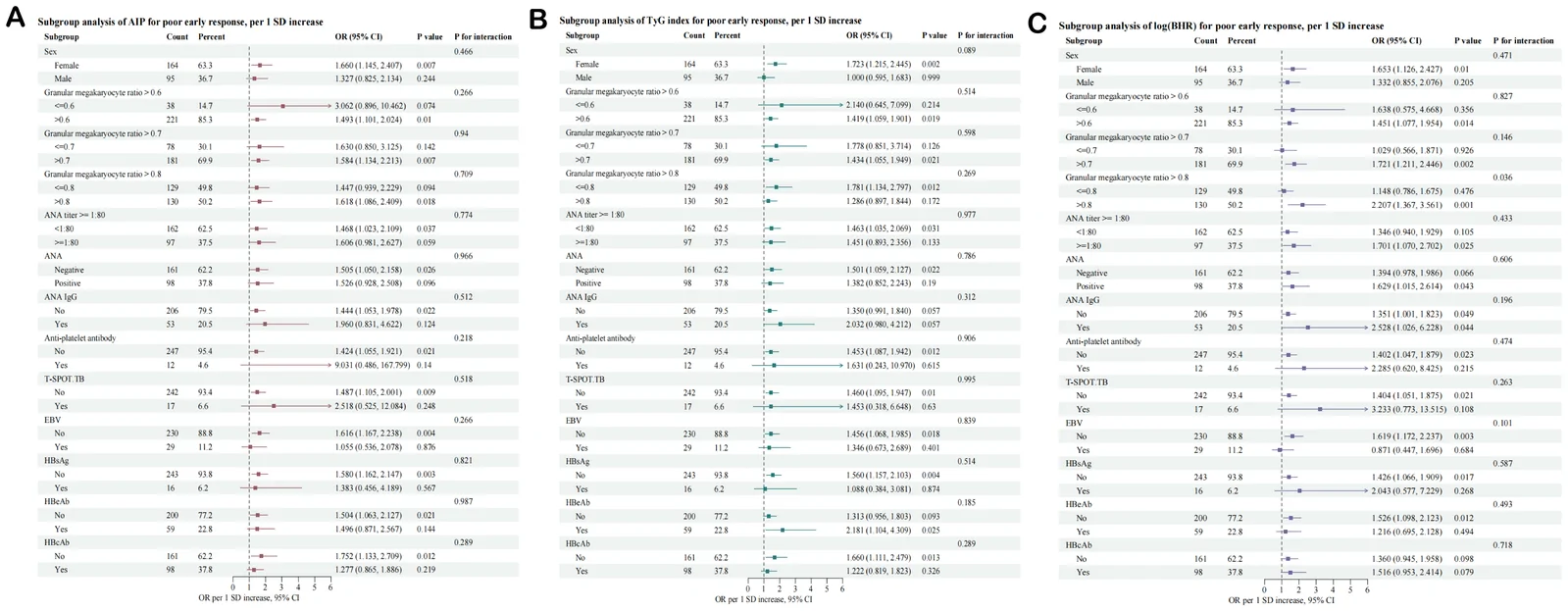
Subgroup analyses of the associations between the derived metabolic indices and poor early response to corticosteroid therapy. Forest plots show the odds ratios and 95% confidence intervals for poor early response at day 7 per 1-standard-deviation increase in **(A)** AIP, **(B)** TyG, and **(C)** logBHR across selected subgroups. Evidence of an interaction was observed between logBHR and the proportion of granular megakaryocytes categorized using a cutoff of 0.8 (*P* for interaction = 0.036). AIP, atherogenic index of plasma; ANA, antinuclear antibody; CI, confidence interval; EBV, Epstein–Barr virus; HBcAb, hepatitis B core antibody; HBeAb, hepatitis B e antibody; HBsAg, hepatitis B surface antigen; IgG, immunoglobulin G; logBHR [log(BHR)], natural logarithm of the blood urea nitrogen-to-high-density lipoprotein cholesterol ratio; OR, odds ratio; SD, standard deviation; T-SPOT.TB, a T-cell-based interferon-γ release assay for tuberculosis; TyG, triglyceride–glucose index.

### 3.5 Discriminatory performance of the derived indices

After multivariable adjustment, the models incorporating AIP, TyG, or logBHR showed limited ability to discriminate patients with poor early response (**Table 3**). The AIP model yielded an area under the receiver operating characteristic curve (AUC) of 0.675 (95% CI, 0.601–0.749), compared with 0.662 (95% CI, 0.588–0.737) for the TyG model and 0.658 (95% CI, 0.586–0.730) for the logBHR model. Overall, the three models showed limited discriminatory performance, with the AIP model yielding a numerically higher AUC than the TyG and logBHR models (**Figure 4**).

**Table 3:** ROC analysis of metabolic-derived index–based prediction models for poor early corticosteroid response.

Model	AUC (95% CI)	*P* value	Cutoff probability	Sensitivity	Specificity	Accuracy	PPV	NPV	Youden index
AIP adjusted model	0.675 (0.601–0.749)	< 0.001	0.276	0.576	0.741	0.699	0.432	0.836	0.317
TyG adjusted model	0.662 (0.588–0.737)	< 0.001	0.206	0.848	0.446	0.548	0.344	0.896	0.294
logBHR adjusted model	0.658 (0.586–0.730)	< 0.001	0.204	0.833	0.446	0.544	0.34	0.887	0.279

P values were calculated using two-sided Wald tests based on DeLong variance estimates under the null hypothesis that the AUC equals 0.50. AIP, atherogenic index of plasma; AUC, area under the receiver operating characteristic curve; CI, confidence interval; logBHR, natural logarithm of the blood urea nitrogen-to-high-density lipoprotein cholesterol ratio; NPV, negative predictive value; PPV, positive predictive value; ROC, receiver operating characteristic; TyG, triglyceride–glucose index.

**Figure 4:**
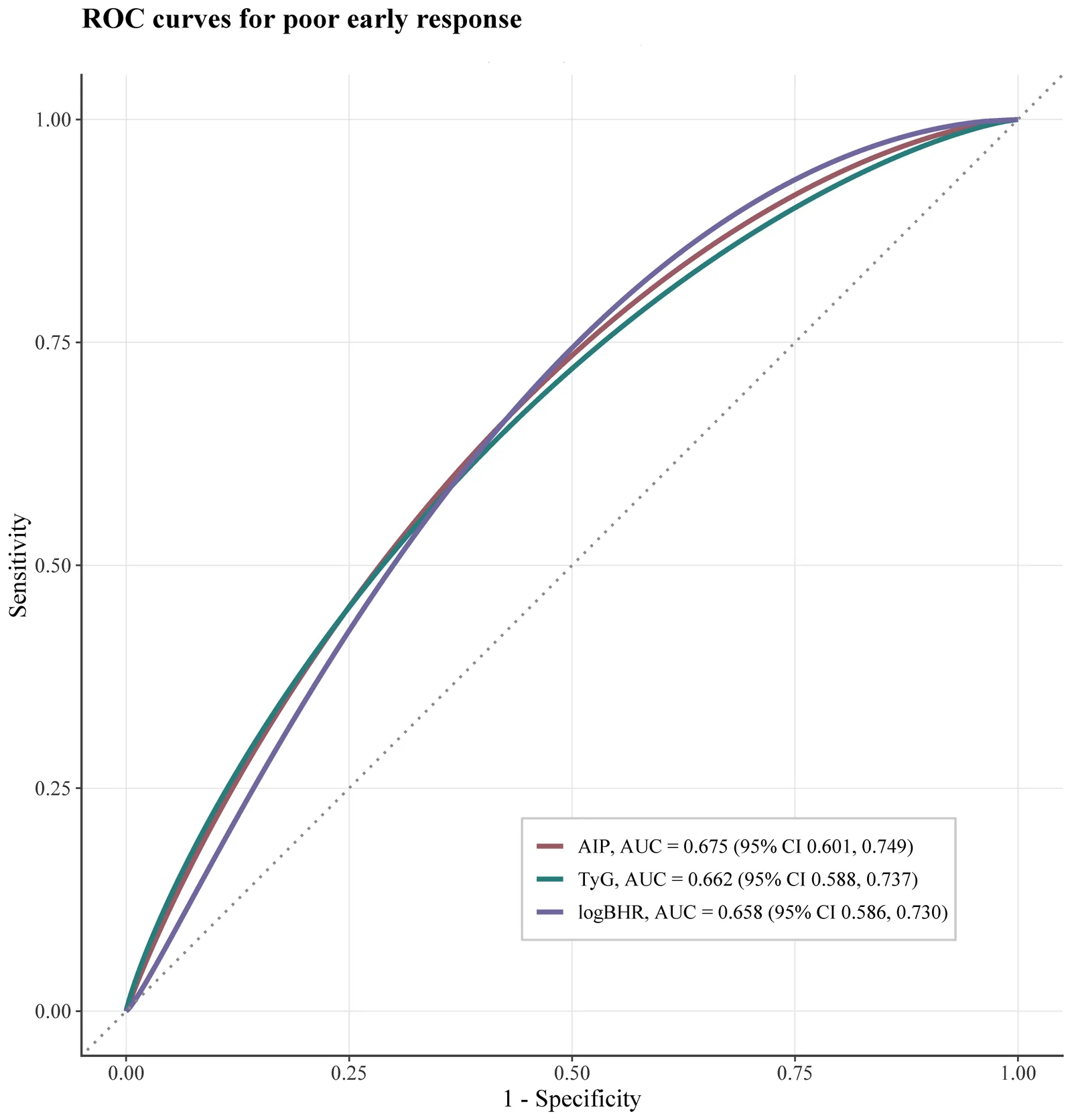
Receiver operating characteristic curves for adjusted models incorporating the derived metabolic indices. Smoothed receiver operating characteristic curves show the ability of logistic regression models incorporating AIP, TyG, or logBHR to discriminate poor early response at day 7. Each model was adjusted for age, sex, EBV status, HBsAg status, and HBeAb status. AIP, atherogenic index of plasma; AUC, area under the receiver operating characteristic curve; CI, confidence interval; EBV, Epstein–Barr virus; HBeAb, hepatitis B e antibody; HBsAg, hepatitis B surface antigen; logBHR, natural logarithm of the blood urea nitrogen-to-high-density lipoprotein cholesterol ratio; ROC, receiver operating characteristic; TyG, triglyceride–glucose index.

## 4 Discussion

This study found that higher pretreatment levels of AIP, TyG, and logBHR were associated with greater odds of nonresponse at day 7 after first-line corticosteroid therapy in adults with primary ITP. The findings were generally consistent across analyses in which the indices were modeled as continuous variables or quartile-based categorical variables, as well as in the restricted cubic spline analyses. Collectively, these findings suggest that indices derived from routine laboratory measurements may reflect metabolic and inflammatory states associated with the early response to corticosteroid therapy in ITP.

The observed associations of AIP, TyG, and logBHR with early corticosteroid response in patients with ITP suggest that metabolic abnormalities may be more than accompanying features of the disease and could contribute to heterogeneity in treatment response. Advances in immunometabolism have shown that immune-cell activation, differentiation, and effector functions are closely regulated by cellular metabolic states. Disturbances in lipid metabolism may therefore contribute to the development and progression of autoimmune diseases by altering immune-cell energy supply and inflammatory signaling (22–24). Lipid abnormalities may influence treatment response in ITP through several pathways. Elevated triglyceride and reduced HDL-C levels may promote oxidative stress and chronic low-grade inflammation, potentially exacerbating platelet destruction (25, 26). Dysregulated lipid metabolism may also impair the restoration of immune tolerance and attenuate the immunosuppressive effects of corticosteroids (24, 27). Previous studies have identified differences in plasma metabolite and lipid profiles associated with glucocorticoid responsiveness in patients with ITP (16, 28). Persistent inflammatory activation and metabolic stress may weaken the anti-inflammatory effects of corticosteroids by altering glucocorticoid receptor signaling and downstream pathways, including the PI3K/Akt/mTOR pathway. These mechanisms may partly explain the poor early response observed in some patients with ITP.

AIP was initially developed primarily for cardiovascular risk assessment (10, 29). However, accumulating evidence indicates that dysfunctional HDL may promote oxidized lipid accumulation, monocyte recruitment, macrophage inflammatory activation, and the release of proinflammatory cytokines (30). A higher AIP may therefore reflect a lipid environment favoring inflammatory activation. In ITP, Fcγ receptor-dependent platelet phagocytosis by macrophages is a major mechanism underlying thrombocytopenia (31). A persistently proinflammatory lipid environment could enhance monocyte–macrophage activation and antibody-mediated platelet clearance, potentially influencing the response to the immunomodulatory effects of corticosteroids (32). Unlike AIP, the TyG index primarily reflects systemic metabolic stress related to impaired glucose and lipid metabolism and insulin resistance (33, 34). Insulin resistance is increasingly recognized not simply as a metabolic disorder but as an immunometabolic state accompanied by chronic inflammatory activation (25, 35). Excess nutrient signaling during insulin resistance can induce reactive oxygen species production and activate inflammatory pathways, including NF-κB, thereby promoting the release of proinflammatory cytokines such as interleukin-6 and tumor necrosis factor-α (36). Immune dysregulation in ITP includes enhanced Th1 and Th17 responses, reduced numbers or impaired function of regulatory T cells, and aberrant B-cell activation with increased autoantibody production (37–39). Higher TyG levels may therefore reflect a metabolic state unfavorable to the restoration of immune tolerance, potentially limiting the ability of corticosteroids to re-establish immune homeostasis. In contrast to conventional lipid measures, BHR integrates information from both BUN and HDL-C and has recently been investigated as a marker of adverse outcomes across several disease settings (14). Elevated BUN may reflect increased protein catabolism, metabolic stress, and an inflammation-related catabolic state, whereas persistent immune activation may contribute to nutritional depletion and metabolic reprogramming (30, 40, 41). Because AIP and TyG share TG as a component, whereas AIP and logBHR share HDL-C, the three indices may be correlated to some extent.

The fully adjusted model additionally included baseline virological markers, including EBV, HBsAg, and HBeAb status, because viral infection or previous viral exposure may influence the immunological background of ITP and platelet levels. Previous research reported a higher prevalence of EBV-DNA positivity among patients with chronic ITP than among healthy controls and found that higher EBV loads were associated with lower platelet counts. These findings suggest that EBV infection or reactivation may contribute to immune dysregulation and platelet destruction in ITP (19). HBV infection may also affect platelet homeostasis through immune-mediated platelet clearance, suppression of bone marrow and megakaryocyte function, hepatic inflammation, and altered regulation of thrombopoietin (42). A large cohort study further showed that HBsAg positivity was independently associated with an increased incidence of thrombocytopenia (20). Viral-associated immune activation may affect both the baseline disease phenotype and the response to immunosuppressive therapy, thereby potentially confounding the associations of the derived metabolic indices with day-7 nonresponse.

The subgroup analyses provided further insight into the associations between the metabolic indices and day-7 nonresponse. No statistically significant interactions were detected for AIP or TyG across the examined subgroups, and most interaction tests for logBHR were also nonsignificant. These findings provided limited evidence of heterogeneity, although they do not establish equivalent associations across subgroups. For example, TyG was significantly associated with nonresponse in women but not in men; however, the interaction test did not establish a sex difference (*P* for interaction = 0.089). Some subgroups defined by positive immunological or viral markers were small, resulting in wide confidence intervals and limiting the precision of subgroup estimates. A notable finding was the stronger association between logBHR and nonresponse in patients with a granular megakaryocyte proportion > 0.8. The OR per 1-SD increase in logBHR was 2.207 (95% CI, 1.367–3.561) in this subgroup, compared with 1.148 (95% CI, 0.786–1.675) in patients with a proportion ≤ 0.8 (*P* for interaction = 0.036). Previous studies have described altered megakaryocyte maturation in ITP and an association between abnormal megakaryocyte morphology and corticosteroid nonresponse (43, 44). These findings suggest that the association between metabolic status and early treatment response may vary by bone marrow phenotype, although the underlying mechanism remains unclear. Given the multiple comparisons and nonsignificant interactions at cutoffs of 0.6 and 0.7, the interaction at 0.8 remains exploratory and requires independent validation before clinical application.

Day-7 nonresponse in this study represents a clinically relevant early response phenotype and should not be equated with corticosteroid resistance, refractory ITP, or definitive treatment failure. The International Working Group on ITP recommends that the timing of response assessment be prespecified according to the expected onset of action of the treatment administered (45, 46). Thus, some patients who do not meet the criteria for complete response or response by day 7 may still achieve a platelet response later. Nevertheless, day-7 nonresponse remains clinically relevant because it may indicate a prolonged period of severe thrombocytopenia and bleeding risk. These patients may require closer monitoring of platelet counts and bleeding manifestations, as well as timely consideration of rescue treatment, such as intravenous immunoglobulin, in the presence of active bleeding, a high risk of bleeding, or an urgent need to increase the platelet count (5). The potential clinical value of these readily available metabolic indices may therefore lie in identifying patients who warrant closer early monitoring, rather than in determining corticosteroid efficacy or directly guiding treatment modification.

The findings are most directly relevant to hospitalized adults with newly diagnosed primary ITP receiving corticosteroid-containing first-line therapy in settings similar to ours. Generalizability to children, patients with secondary or persistent/chronic ITP, and outpatient populations remains uncertain. Differences in disease severity, metabolic comorbidities, and treatment practices across centers may also affect these associations, highlighting the need for validation in broader populations.

This study has several limitations. First, its single-center retrospective design may have introduced selection bias, information bias, and unmeasured confounding. Excluding patients with missing key variables may also have resulted in complete-case selection bias. Second, the use of a single baseline AIP measurement limited our assessment of within-person variability; serial measurements would help determine whether changes in AIP provide additional information on treatment response. Third, the regression models did not adjust for corticosteroid regimen, dose, or concomitant therapy. Differences in treatment may have influenced day-7 response status and the observed associations. Consequently, the day-7 outcome reflects early response under the treatment strategies used at our center. Fourth, the number and scope of covariates included in the fully adjusted model were limited. Residual confounding may therefore remain because of factors such as renal function, baseline platelet count, bleeding severity, metabolic comorbidities, and concomitant medications. Fifth, the 66 nonresponse events limited the precision of the multivariable models, restricted cubic spline analyses, and subgroup analyses. The evaluation of multiple indices, parameterizations, and subgroups also increased the likelihood of chance findings. Finally, the ROC results represent apparent performance estimated from the same dataset, without correction for optimism through internal validation or confirmation in an external cohort.

In this single-center retrospective cohort, higher pretreatment levels of AIP, TyG, and logBHR were associated with greater odds of day-7 nonresponse among adults with primary ITP receiving corticosteroid-containing first-line therapy. However, these findings should be considered exploratory and do not support the use of these indices alone to guide treatment decisions. These associations require validation in independent prospective cohorts with standardized treatment protocols and outcome assessments, together with evaluation of their incremental value beyond established clinical factors.

## Data Availability

The original contributions presented in the study are included in the article/**Supplementary Material**. Further inquiries can be directed to the corresponding author.
