# Supplementary material for: Derived metabolic indices and poor early response to corticosteroids in adults with primary immune thrombocytopenia: a retrospective cohort study
Source: Front Endocrinol (Lausanne). 2026 Oct 28;17:1981987. doi: 10.3389/fendo.2026.1981987 (PMC13752056; doi:10.3389/fendo.2026.1981987)
Supplement: Supplementary file 1 [file DataSheet1.pdf]

Supplementary regression analyses

Complete logistic regression results for AIP as a continuous variable

Characteristic	Model 1 (N = 259)		Model 2 (N = 259)		Model 3 (N = 259)	
	OR (95% CI)	*P* value	OR (95% CI)	*P* value	OR (95% CI)	*P* value
AIP	5.554 (1.711–18.940)	0.005	5.422 (1.658–18.610)	0.006	5.726 (1.711–20.150)	0.005
Age	—	—	1.013 (0.995–1.031)	0.167	1.013 (0.994–1.032)	0.185
**Sex**						
Female	—	—	Ref	—	Ref	—
Male	—	—	0.808 (0.438–1.460)	0.485	0.834 (0.448–1.523)	0.559
**EBV**						
Negative	—	—	—	—	Ref	—
Positive	—	—	—	—	1.441 (0.590–3.341)	0.405
**HBsAg**						
Negative	—	—	—	—	Ref	—
Positive	—	—	—	—	2.947 (0.953–8.852)	0.054
**HBeAb**						
Negative	—	—	—	—	Ref	—
Positive	—	—	—	—	1.434 (0.730–2.758)	0.286

Outcome: poor early response at day 7 (EARLY_RESPONSE = 1). Model 1 was unadjusted. Model 2 was adjusted for age and sex. Model 3 was additionally adjusted for EBV, HBsAg, and HBeAb.

ORs for AIP and age are reported per 1-unit and 1-year increase, respectively.

Abbreviations: AIP, atherogenic index of plasma; CI, confidence interval; EBV, Epstein–Barr virus; HBeAb, hepatitis B e antibody; HBsAg, hepatitis B surface antigen; N, number of patients; OR, odds ratio.

Complete logistic regression results for AIP using quartile medians for trend analysis

Characteristic	Model 1 (N = 259)		Model 2 (N = 259)		Model 3 (N = 259)	
	OR (95% CI)	*P* value	OR (95% CI)	*P* value	OR (95% CI)	*P* value
AIP (quartile median)	6.650 (1.596–29.020)	0.010	6.408 (1.517–28.290)	0.013	7.183 (1.650–32.870)	0.010
Age	—	—	1.013 (0.995–1.031)	0.173	1.012 (0.994–1.032)	0.203
**Sex**						
Female	—	—	Ref	—	Ref	—
Male	—	—	0.807 (0.438–1.456)	0.482	0.834 (0.449–1.522)	0.559
**EBV**						
Negative	—	—	—	—	Ref	—
Positive	—	—	—	—	1.515 (0.625–3.495)	0.340
**HBsAg**						
Negative	—	—	—	—	Ref	—
Positive	—	—	—	—	2.916 (0.943–8.772)	0.056
**HBeAb**						
Negative	—	—	—	—	Ref	—
Positive	—	—	—	—	1.472 (0.750–2.826)	0.251

Outcome: poor early response at day 7 (EARLY_RESPONSE = 1). Model 1 was unadjusted. Model 2 was adjusted for age and sex. Model 3 was additionally adjusted for EBV, HBsAg, and HBeAb.

The median AIP value within each quartile was assigned to patients in that quartile and modeled continuously. The P value for this term is the P for trend. ORs are per 1-unit increase in the assigned median and per 1-year increase in age.

Abbreviations: AIP, atherogenic index of plasma; CI, confidence interval; EBV, Epstein–Barr virus; HBeAb, hepatitis B e antibody; HBsAg, hepatitis B surface antigen; N, number of patients; OR, odds ratio.

Complete logistic regression results for AIP by quartile

Characteristic	Model 1 (N = 259)		Model 2 (N = 259)		Model 3 (N = 259)	
	OR (95% CI)	*P* value	OR (95% CI)	*P* value	OR (95% CI)	*P* value
**AIP (quartiles)**						
Q1	Ref	—	Ref	—	Ref	—
Q2	1.796 (0.755–4.448)	0.192	1.822 (0.759–4.551)	0.185	2.000 (0.818–5.109)	0.135
Q3	1.989 (0.843–4.905)	0.122	1.989 (0.839–4.928)	0.125	2.087 (0.865–5.276)	0.108
Q4	3.012 (1.325–7.263)	0.010	2.974 (1.295–7.238)	0.012	3.247 (1.384–8.105)	0.009
Age	—	—	1.012 (0.995–1.031)	0.176	1.012 (0.994–1.031)	0.208
**Sex**						
Female	—	—	Ref	—	Ref	—
Male	—	—	0.795 (0.431–1.438)	0.453	0.819 (0.440–1.497)	0.522
**EBV**						
Negative	—	—	—	—	Ref	—
Positive	—	—	—	—	1.527 (0.629–3.528)	0.332
**HBsAg**						
Negative	—	—	—	—	Ref	—
Positive	—	—	—	—	2.939 (0.948–8.892)	0.055
**HBeAb**						
Negative	—	—	—	—	Ref	—
Positive	—	—	—	—	1.504 (0.764–2.900)	0.228

Outcome: poor early response at day 7 (EARLY_RESPONSE = 1). Model 1 was unadjusted. Model 2 was adjusted for age and sex. Model 3 was additionally adjusted for EBV, HBsAg, and HBeAb.

Q1 is the reference quartile. The OR for age is reported per 1-year increase.

Abbreviations: AIP, atherogenic index of plasma; CI, confidence interval; EBV, Epstein–Barr virus; HBeAb, hepatitis B e antibody; HBsAg, hepatitis B surface antigen; N, number of patients; OR, odds ratio; Q, quartile.

Complete logistic regression results for TyG as a continuous variable

Characteristic	Model 1 (N = 259)		Model 2 (N = 259)		Model 3 (N = 259)	
	OR (95% CI)	*P* value	OR (95% CI)	*P* value	OR (95% CI)	*P* value
TyG	2.102 (1.213–3.707)	0.009	1.998 (1.146–3.543)	0.016	2.105 (1.194–3.780)	0.011
Age	—	—	1.011 (0.993–1.029)	0.245	1.010 (0.992–1.030)	0.284
**Sex**						
Female	—	—	Ref	—	Ref	—
Male	—	—	0.850 (0.463–1.531)	0.592	0.883 (0.477–1.606)	0.687
**EBV**						
Negative	—	—	—	—	Ref	—
Positive	—	—	—	—	1.470 (0.604–3.398)	0.378
**HBsAg**						
Negative	—	—	—	—	Ref	—
Positive	—	—	—	—	3.013 (0.969–9.107)	0.050
**HBeAb**						
Negative	—	—	—	—	Ref	—
Positive	—	—	—	—	1.477 (0.752–2.835)	0.247

Outcome: poor early response at day 7 (EARLY_RESPONSE = 1). Model 1 was unadjusted. Model 2 was adjusted for age and sex. Model 3 was additionally adjusted for EBV, HBsAg, and HBeAb.

ORs for TyG and age are reported per 1-unit and 1-year increase, respectively.

Abbreviations: CI, confidence interval; EBV, Epstein–Barr virus; HBeAb, hepatitis B e antibody; HBsAg, hepatitis B surface antigen; N, number of patients; OR, odds ratio; TyG, triglyceride–glucose index.

Complete logistic regression results for TyG using quartile medians for trend analysis

Characteristic	Model 1 (N = 259)		Model 2 (N = 259)		Model 3 (N = 259)	
	OR (95% CI)	*P* value	OR (95% CI)	*P* value	OR (95% CI)	*P* value
TyG (quartile median)	2.460 (1.255–4.893)	0.009	2.328 (1.180–4.661)	0.016	2.442 (1.220–4.971)	0.012
Age	—	—	1.011 (0.993–1.030)	0.232	1.010 (0.992–1.030)	0.276
**Sex**						
Female	—	—	Ref	—	Ref	—
Male	—	—	0.837 (0.456–1.509)	0.559	0.868 (0.468–1.579)	0.646
**EBV**						
Negative	—	—	—	—	Ref	—
Positive	—	—	—	—	1.540 (0.636–3.550)	0.320
**HBsAg**						
Negative	—	—	—	—	Ref	—
Positive	—	—	—	—	2.966 (0.951–8.998)	0.054
**HBeAb**						
Negative	—	—	—	—	Ref	—
Positive	—	—	—	—	1.430 (0.727–2.748)	0.290

Outcome: poor early response at day 7 (EARLY_RESPONSE = 1). Model 1 was unadjusted. Model 2 was adjusted for age and sex. Model 3 was additionally adjusted for EBV, HBsAg, and HBeAb.

The median TyG value within each quartile was assigned to patients in that quartile and modeled continuously. The P value for this term is the P for trend. ORs are per 1-unit increase in the assigned median and per 1-year increase in age.

Abbreviations: CI, confidence interval; EBV, Epstein–Barr virus; HBeAb, hepatitis B e antibody; HBsAg, hepatitis B surface antigen; N, number of patients; OR, odds ratio; TyG, triglyceride–glucose index.

Complete logistic regression results for TyG by quartile

Characteristic	Model 1 (N = 259)		Model 2 (N = 259)		Model 3 (N = 259)	
	OR (95% CI)	*P* value	OR (95% CI)	*P* value	OR (95% CI)	*P* value
**TyG (quartiles)**						
Q1	Ref	—	Ref	—	Ref	—
Q2	0.815 (0.330–1.983)	0.652	0.811 (0.327–1.978)	0.644	0.885 (0.351–2.212)	0.794
Q3	1.565 (0.696–3.599)	0.282	1.600 (0.707–3.704)	0.262	1.757 (0.756–4.205)	0.195
Q4	2.341 (1.077–5.272)	0.035	2.200 (1.001–5.001)	0.053	2.363 (1.051–5.531)	0.041
Age	—	—	1.011 (0.993–1.030)	0.227	1.011 (0.992–1.030)	0.266
**Sex**						
Female	—	—	Ref	—	Ref	—
Male	—	—	0.832 (0.451–1.507)	0.550	0.862 (0.464–1.575)	0.633
**EBV**						
Negative	—	—	—	—	Ref	—
Positive	—	—	—	—	1.527 (0.629–3.531)	0.332
**HBsAg**						
Negative	—	—	—	—	Ref	—
Positive	—	—	—	—	2.876 (0.914–8.828)	0.064
**HBeAb**						
Negative	—	—	—	—	Ref	—
Positive	—	—	—	—	1.430 (0.724–2.758)	0.293

Outcome: poor early response at day 7 (EARLY_RESPONSE = 1). Model 1 was unadjusted. Model 2 was adjusted for age and sex. Model 3 was additionally adjusted for EBV, HBsAg, and HBeAb.

Q1 is the reference quartile. The OR for age is reported per 1-year increase.

Abbreviations: CI, confidence interval; EBV, Epstein–Barr virus; HBeAb, hepatitis B e antibody; HBsAg, hepatitis B surface antigen; N, number of patients; OR, odds ratio; Q, quartile; TyG, triglyceride–glucose index.

Complete logistic regression results for logBHR as a continuous variable

Characteristic	Model 1 (N = 259)		Model 2 (N = 259)		Model 3 (N = 259)	
	OR (95% CI)	*P* value	OR (95% CI)	*P* value	OR (95% CI)	*P* value
logBHR	2.584 (1.289–5.316)	0.008	2.562 (1.221–5.504)	0.014	2.331 (1.093–5.075)	0.030
Age	—	—	1.008 (0.990–1.027)	0.407	1.008 (0.989–1.028)	0.410
**Sex**						
Female	—	—	Ref	—	Ref	—
Male	—	—	0.735 (0.393–1.341)	0.323	0.774 (0.412–1.426)	0.418
**EBV**						
Negative	—	—	—	—	Ref	—
Positive	—	—	—	—	1.412 (0.575–3.286)	0.434
**HBsAg**						
Negative	—	—	—	—	Ref	—
Positive	—	—	—	—	2.296 (0.759–6.690)	0.128
**HBeAb**						
Negative	—	—	—	—	Ref	—
Positive	—	—	—	—	1.433 (0.731–2.746)	0.284

Outcome: poor early response at day 7 (EARLY_RESPONSE = 1). Model 1 was unadjusted. Model 2 was adjusted for age and sex. Model 3 was additionally adjusted for EBV, HBsAg, and HBeAb.

ORs for logBHR and age are reported per 1-unit and 1-year increase, respectively.

Abbreviations: CI, confidence interval; EBV, Epstein–Barr virus; HBeAb, hepatitis B e antibody; HBsAg, hepatitis B surface antigen; logBHR, natural logarithm of the blood urea nitrogen-to-high-density lipoprotein cholesterol ratio; N, number of patients; OR, odds ratio.

Complete logistic regression results for logBHR using quartile medians for trend analysis

Characteristic	Model 1 (N = 259)		Model 2 (N = 259)		Model 3 (N = 259)	
	OR (95% CI)	*P* value	OR (95% CI)	*P* value	OR (95% CI)	*P* value
logBHR (quartile median)	5.543 (2.242–14.230)	<0.001	5.598 (2.166–15.040)	<0.001	5.066 (1.933–13.770)	0.001
Age	—	—	1.006 (0.988–1.025)	0.537	1.006 (0.987–1.025)	0.542
**Sex**						
Female	—	—	Ref	—	Ref	—
Male	—	—	0.720 (0.385–1.316)	0.293	0.752 (0.399–1.387)	0.369
**EBV**						
Negative	—	—	—	—	Ref	—
Positive	—	—	—	—	1.425 (0.574–3.358)	0.428
**HBsAg**						
Negative	—	—	—	—	Ref	—
Positive	—	—	—	—	2.306 (0.755–6.791)	0.130
**HBeAb**						
Negative	—	—	—	—	Ref	—
Positive	—	—	—	—	1.359 (0.687–2.628)	0.369

Outcome: poor early response at day 7 (EARLY_RESPONSE = 1). Model 1 was unadjusted. Model 2 was adjusted for age and sex. Model 3 was additionally adjusted for EBV, HBsAg, and HBeAb.

The median logBHR value within each quartile was assigned to patients in that quartile and modeled continuously. The P value for this term is the P for trend. ORs are per 1-unit increase in the assigned median and per 1-year increase in age.

Abbreviations: CI, confidence interval; EBV, Epstein–Barr virus; HBeAb, hepatitis B e antibody; HBsAg, hepatitis B surface antigen; logBHR, natural logarithm of the blood urea nitrogen-to-high-density lipoprotein cholesterol ratio; N, number of patients; OR, odds ratio.

Supplementary Table S10

Complete logistic regression results for logBHR by quartile

Characteristic	Model 1 (N = 259)		Model 2 (N = 259)		Model 3 (N = 259)	
	OR (95% CI)	P value	OR (95% CI)	P value	OR (95% CI)	P value
**logBHR (quartiles)**						
Q1	Reference	—	Reference	—	Reference	—
Q2	1.956 (0.773–5.255)	0.165	1.995 (0.780–5.415)	0.158	1.795 (0.689–4.931)	0.238
Q3	2.788 (1.144–7.333)	0.029	2.895 (1.168–7.728)	0.026	2.819 (1.130–7.567)	0.031
Q4	4.750 (2.022–12.230)	<0.001	4.825 (1.967–12.920)	<0.001	4.272 (1.716–11.560)	0.003
Age	—	—	1.006 (0.988–1.025)	0.540	1.006 (0.987–1.025)	0.537
**Sex**						
Female	—	—	Reference	—	Reference	—
Male	—	—	0.714 (0.383–1.305)	0.281	0.747 (0.397–1.377)	0.356
**EBV**						
Negative	—	—	—	—	Reference	—
Positive	—	—	—	—	1.448 (0.582–3.426)	0.409
**HBsAg**						
Negative	—	—	—	—	Reference	—
Positive	—	—	—	—	2.304 (0.752–6.823)	0.132
**HBeAb**						
Negative	—	—	—	—	Reference	—
Positive	—	—	—	—	1.361 (0.687–2.635)	0.367

Outcome: poor early response at day 7 (EARLY_RESPONSE = 1). Model 1 was unadjusted. Model 2 was adjusted for age and sex. Model 3 was additionally adjusted for EBV, HBsAg, and HBeAb.

Q1 is the reference quartile. The OR for age is reported per 1-year increase.

Reference indicates the reference category; — indicates a term not included in that model or a statistic not applicable to a reference category.

Abbreviations: CI, confidence interval; EBV, Epstein–Barr virus; HBeAb, hepatitis B e antibody; HBsAg, hepatitis B surface antigen; logBHR, natural logarithm of the blood urea nitrogen-to-high-density lipoprotein cholesterol ratio; N, number of patients; OR, odds ratio; Q, quartile.
